# Bilateral Parotid Gland Cysts as an Atypical Indicator of Sjögren's Syndrome: A Case Study and Literature Overview

**DOI:** 10.22038/ijorl.2025.85419.3869

**Published:** 2025

**Authors:** Stefania Troise, Giuseppe Tarallo, Emanuele Carraturo, Fabio Di Blasi, Marco Sarcinella, Maria Esposito, Federica Calabria, Iaquino Vincenzo, Giovanni Dell’Aversana Orabona

**Affiliations:** 1 *Maxillofacial Surgery Unit, Department of Neurosciences, Reproductive and Odontostomatological Sciences, University of Naples Federico II, Naples, Italy.*

**Keywords:** Autoimmune salivary gland disorder, Sjögren's syndrome, Bilateral parotid cysts, Case report, Literature review

## Abstract

**Introduction:**

Sjögren's syndrome is a systemic autoimmune disorder that gradually impairs exocrine function, primarily affecting lacrimal and salivary glands.

**Case Report:**

We describe an unusual presentation involving an elderly female patient diagnosed with late-stage Sjögren's syndrome. Laboratory testing eliminated viral infections including HIV and HCV. Fine-needle aspiration biopsy of parotid swellings revealed inflammatory cystic lesions, excluding malignancy and other common cystic conditions. MRI revealed several fluid-filled nodules in both glands.

**Conclusion:**

This report supports including Sjögren's syndrome in the differential diagnosis for bilateral cystic lesions of the parotid glands. A synthesis of similar literature cases is included.

## Introduction

Sjögren's syndrome (SS) is a chronic autoimmune disease which the patient’s immune system primarily targets moisture-producing glands, particularly the lacrimal and salivary tissues (1).

The autoimmune insult reduces glandular secretions, leading to common symptoms such as dry mouth (xerostomia) and dry eyes (keratoconjunctivitis sicca), but SS may also affect systemic organs including joints, lungs, skin, kidneys, and the peripheral nervous system (1,2).

The disease manifests variably, with hallmark symptoms such as ocular and oral dryness, swallowing difficulties, hoarseness, joint discomfort, fatigue, skin issues, and respiratory symptoms.

Diagnosing SS involves clinical evaluation, laboratory screening for characteristic antibodies, functional glandular testing, imaging, and frequently biopsy confirmation (2,3). Although not routinely used, advanced imaging like MRI may incidentally detect parotid cysts in atypical SS presentations (4). This report documents such a presentation and compares it to cases previously described in scientific literature.

## Case Report

This case was managed at the Maxillofacial Surgery Unit of "Federico II" University Hospital, as part of a broader review.

**Fig 1:**
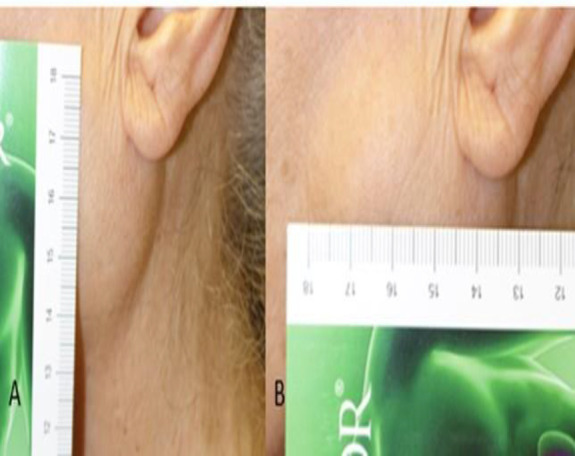
Dimensions of the left parotid mass displayed in sagittal (B) and vertical (A) MRI slices.

An 81-year-old woman presented with bilateral swelling localized in the parotid region. Physical assessment revealed a 4 x 4.5 cm lesion in the left parotid gland (Figure 1).

**Fig 2:**
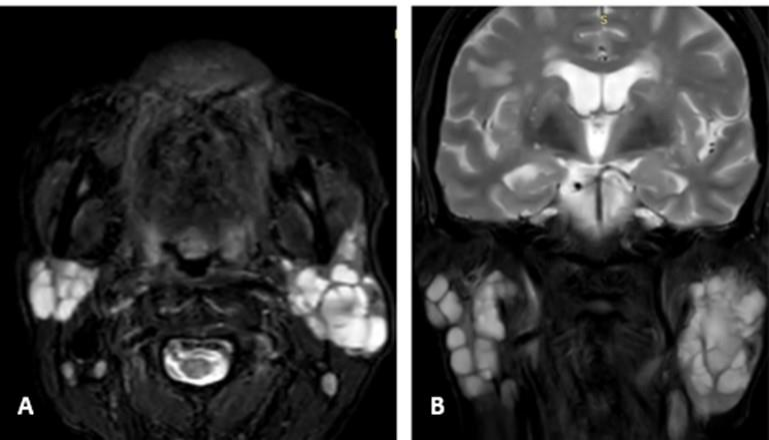
T2-weighted axial (A) and coronal (B) MRI images demonstrating multiple, partially confluent cysts in both parotid glands.

The patient reported no discomfort, and facial nerve function was intact. She had been previously diagnosed with untreated grade IV Sjögren's syndrome. Viral screenings for HIV and HCV returned negative. Cytological analysis using fine-needle aspiration indicated cystic structures with inflammatory infiltration, excluding diagnoses such as Warthin tumour, mucoceles, lymphoepithelial cysts, and malignancy. MRI scans identified multiple fluid-filled cysts in both parotid glands, with the largest reaching 18 mm on the left side (Figure 2). Further serological evaluation showed raised Anti-Ro/SSA, Anti-La/SSB, ANA, and rheumatoid factor levels. No HIV-related markers were detected.

Due to her comorbidities, age, and lack of symptoms, surgical treatment was not pursued. The patient was monitored over a six-month period during which her condition remained stable.

## Discussion

Sjögren's syndrome (SS) is a multi-system immune-mediated disease targeting exocrine organs such as the salivary and lacrimal glands. Defining features include dryness (sicca symptoms), systemic manifestations, lymphocytic infiltration, autoantibody presence, and a higher probability of lymphoma. The condition affects women disproportionately and is commonly seen alongside other autoimmune diseases like lupus (14%), rheumatoid arthritis (17%), and systemic sclerosis (12%) (1,2).

Potential complications include malignancy (particularly lymphomas), interstitial lung disease, nephritis, and vasculitis. Triggers may include genetic factors and environmental stimuli such as Epstein-Barr Virus (EBV) and Human Herpesvirus 6 (HHV-6), which may activate salivary epithelial cells to express self-antigens (3).

Symptoms can be classified into gland-specific (dry mouth, parotid swelling) and systemic (fatigue, fever, weight loss). Diagnosis often involves a combination of autoantibody detection, salivary flow evaluation, and biopsy of minor salivary glands, showing lymphocytic aggregates (4).

Therapeutic approaches are centered around symptom control and immune modulation using conventional DMARDs and biologics like hydroxychloroquine, methotrexate, rituximab, belimumab, or anakinra (4-8).

Though usually presenting with sicca symptoms, SS may sometimes lead to unusual findings such as multicystic parotid changes. In such cases, non-operative management is typically preferred unless complications arise (9-12).

We identified several analogous cases in literature where patients exhibited parotid cysts in the absence of HIV infection. Most cases were managed conservatively due to risks associated with surgery near facial nerve structures. Only Janus et al. opted for surgical resection (13-16).

## Conclusion

This report affirms that multiple bilateral cystic lesions in the parotid glands may signify underlying Sjögren's syndrome. These abnormalities typically exhibit benign behaviour and may be managed through medical therapy and consistent observation, avoiding unnecessary surgical risks.
